# Coronary deaths during Midsummer festival in Finland: miseries of long, light nights

**DOI:** 10.1007/s10654-021-00744-6

**Published:** 2021-04-21

**Authors:** Simo Näyhä

**Affiliations:** Center for Environmental and Respiratory Health Research, University of Oulu, P.O. Box 5000, FI-90014 Oulu, Finland

**Keywords:** Coronary heart disease, Mortality, Alcohol, Holidays, Midsummer

## Abstract

This paper examines whether the anomalous summer peak in deaths from coronary heart disease (CHD) in Finland could be attributed to adverse effects of the Midsummer festival and alcohol consumption during the festival. Daily deaths from CHD and alcohol poisoning in Finland, 1961–2014, that occurred during the 7 days centering on Midsummer Day were analysed in relation to deaths during 14 to 4 days before and 4 to 14 after Midsummer Day. Daily counts of deaths from CHD among persons aged 35–64 years were regressed on days around the Midsummer period by negative binomial regression. Mortality from CHD was highest on Midsummer Day (RR 1.25 (95% confidence interval 1.12–1.31), one day after the peak in deaths from alcohol poisonings. RR for CHD on Midsummer Day was particulary high (RR = 1.43; 1.09–1.86) in the 2000s, 30% of deaths being attributable to that day. In conclusion, the anomalous and prominent summer peak in deaths from CHD in Finland is an adverse consequence of the Midsummer festival. The most likely underlying reason is heavy alcohol consumption during the festival period, especially on Midsummer Eve. In the 2000s, one third of deaths from CHD on Midsummer Day are preventable.

## Introduction

Mortality from coronary heart disease (CHD) among the working-aged population in Finland shows an anomalous bi-seasonal pattern with a conspicuous peak in early summer (June) along with a second peak in winter [1]. There is only one incidental report of a similar seasonal pattern from the Nordic countries [2]. An effect of summer heat is unlikely, since the mean temperature in June in Finland is no higher than 15 ºC, which is the temperature of lowest mortality in this area [3]. Deaths from CHD were therefore analysed on a daily basis to see if the high June mortality could be attributed to the Midsummer festival (Saturday between June 20th and 26^th^) and especially to the heavy alcohol consumption commonly associated with it [4]. In case such an association exists, it would offer an opportunity for prevention.

### Methods

The daily counts of deaths among persons aged 35–64 years in Finland during the period 1961–2014 in which CHD had been recorded as the underlying cause were obtained from Statistics Finland. CHD had been coded as ICD 7 code 420 in 1961–1968, ICD 8 codes 410–414 in 1969–1986, ICD 9 codes 410–414 in 1987–1995 and ICD 10 codes I20-I25 in 1996–2014. Daily deaths from CHD were compared with those from accidental alcohol poisonings (coded respectively as E880, E860, E851 and X45), which were used as an indicator of heavy alcohol consumption. The diagnosis of CHD was based on a medical or forensic autopsy in 31%, 47%, 56%, 68% and 83% of the deaths in the 1960s, 1970s, 1980s 1990s and 2000s, respectively. The diagnosis of alcohol poisoning was based on autopsy in practically all cases. Only deaths among persons resident in Finland were included. The respective populations were obtained from population statistics.

Daily mortality rates and their 95% confidence intervals (CI) were first calculated for all 29 day periods from 14 days before to 14 days after Midsummer Day. The 29 day period was selected because it was short enough to rule out any seasonal effects. The counts of deaths on − 3 to + 3 days around the Midsummer Day were compared with daily counts during − 14 to − 4 and + 4 to + 14 days around the Midsummer Day which was considered as a period long enough to serve as reference. Negative binomial regression was used to allow for extra-Poisson variation. The results were expressed in terms of mortality rates per 1000,000 person-days, rate ratios, rate differences and fractions attributable to each day. Since Midsummer Day is a Saturday, which in itself increases mortality from CHD [1], an adjustment was made for weekdays. To assess the effect of the steep decline of CHD mortality in Finland since the 1970s, an adjustment was also made for time periods (in classes 1961–1969, 1970–1979, 1980–1989, 1990–1999 and 2000–2014) and was complemented by a time-stratified analysis. The entire seasonal pattern of CHD over the year was shown in terms of weekly mortality rates. The calculations were performed using R software, version 3.50 (http://www.Rproject.org).

### Results

**Fig. 1:**
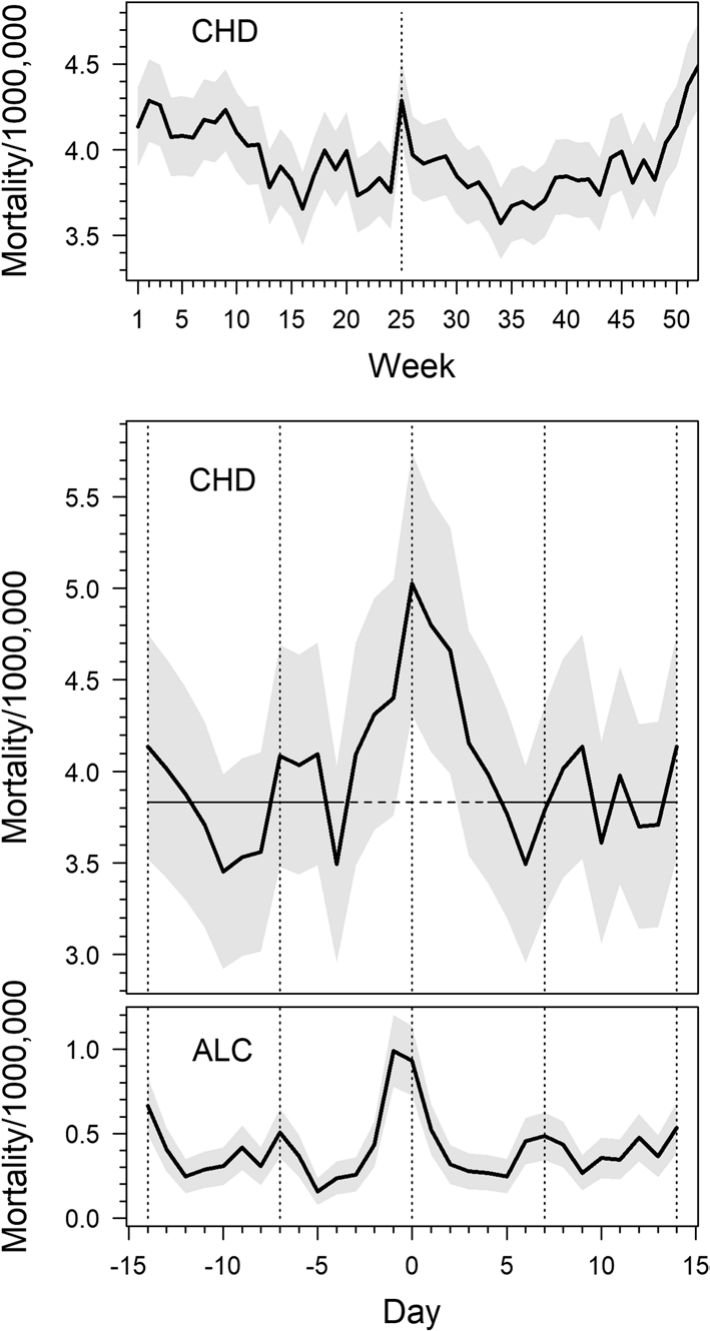
The upper figure shows weekly mortality from coronary heart disease (CHD) during an average year and the two lower ones daily mortalities from CHD and alcohol poisoning (ALC) during Midsummer Day ± 14 days (Midsummer Day marked by 0). Shaded areas indicate 95% confidence bands. The horizontal line shows mortality from CHD during the reference period. Men and women aged 35–64 years, 1961–2014. All mortalities expressed as deaths per 1000,000 person-days

The upper part of Fig. 1 shows that mortality from CHD increased sharply in late June (week 25) and was followed by a winter peak in December (week 52). A close-up of daily mortality on ± 14 days around Midsummer Day (two lower parts of Fig. 1) shows a marked increase in coronary deaths on Midsummer Day, while deaths from alcohol poisoning increased one day before that, i.e. on Midsummer Eve. Compared with mortality during the reference periods (3.83 per 1000,000 person-days), mortality increased consistently up to 5.03/1000,000 (by 31%) on Midsummer Day and declined consistently towards the end of the holiday period (Table 1). Mortality adjusted for weekdays and time periods still showed an excess of 25% on Midsummer Day. Adjusted mortality on Midsummer Eve and two days before that also exceeded the reference rate, as did mortality on three days following the Midsummer Day.

**Table 1:** Mortality from coronary heart disease around Midsummer holiday, 1961–2014

	No. of deaths		Mortality/1000,0000 person-days	RD/1000,000 person-days	Crude RR	Adjusted^a^ RR	Attributable fraction (%) (crude/adjusted)
	Sum	Mean/day					
Reference period^b^	8523	7.17	3.83 (3.71–3.96)	0	1.00	1.00	
Whole Midsummer period^c^	3179	8.41	4.49 (4.25–4.74)	0.66 (0.54–0.79)	1.17 (1.10–1.25)	1.18 (1.13–1.23)	14.7/15.0
Wednesday before Midsummer	414	7.67	4.10 (3.49–4.71)	0.26 (− 0.22–1.00)	1.07 (0.92–1.25)	1.08 (0.96–1.21)	6.5/7.1
Thursday before Midsummer	436	8.07	4.31 (3.68–4.95)	0.48 (− 0.03–1.24)	1.13 (0.97–1.31)	1.18 (1.05–1.32)	11.1/14.9
Friday, Midsummer Eve	445	8.24	4.40 (3.76–5.05)	0.57 (0.05–1.34)	1.15 (0.99–1.34)	1.23 (1.09–1.37)	13.0 /18.5
Saturday, Midsummer Day	508	9.41	5.03 (4.31–5.75)	1.19 (0.60–2.04)	1.31 (1.13–1.52)	1.25 (1.12–1.38)	23.7 /19.8
Sunday after Midsummer	485	8.98	4.80 (4.11–5.49)	0.97 (0.40–1.78)	1.25 (1.08–1.45)	1.19 (1.07–1.33)	20.1/16.0
Monday after Midsummer	471	8.72	4.66 (3.98–5.34)	0.83 (0.27–1.63)	1.22 (1.05–1.41)	1.16 (1.04–1.29)	17.8/13.5
Tuesday after Midsummer	420	7.78	4.16 (3.54–4.77)	0.32 (− 0.17–1.06)	1.08 (0.93–1.26)	1.15 (1.03–1.29)	7.8/13.2
Midsummer Day ± 14 days	11,702	7.47	3.99				

Risk difference (RD) and crude and adjusted risk ratios (RR) relative to the reference period, and attributable fraction of deaths by day

^a^Adjusted for weekdays and time periods

^b^14 to 4 days before and 4 to 14 days after Midsummer Day

^c^Three days before and three days after Midsummer Day

One fifth of the deaths that occurred on Midsummer Day were attributable to that day itself, independently of it being a Saturday (Table 1). The respective fractions attributable to other days during the holiday period ranged from 7 to 19%, the fraction being 15% for the entire 7 day Midsummer period.

There was a sharp decline in CHD mortality since the 1970s, from 7.23 to 1.75 per 1000,000 person-days in the 2000s (Table 2). Consistently with that, the absolute risk attributable to the Midsummer period declined from 1.45 to 0.19 per 1000,000 person-days, the relative excess reducing from 21 to 12%. Along with declining mortality, the relative excess mortality on Midsummer Day remained elevated, especially since the 1980s (40% to 76%). In the 2000s, 30% of the deaths on Midsummer Day were attributable to that special day.

**Table 2:** Mortality from coronary heart disease around Midsummer holiday by periods

Days	No. of deaths		Mortality/1000,0000 person-days	RD/1000,000 person-days	Crude RR	Adjusted^a^ RR	Attributable fraction (%) (crude/adjusted)
	Sum	Mean/day					
1961–1969							
Reference period^b^	1944	9.82	6.34 (6.04–6.64)	0	1	1	
Midsummer period^c^	702	11.14	7.20 (6.63–7.76)	0.86 (0.59–1.12)	1.14 (1.04–1.24)	1.14 (1.04–1.25)	11.9/12.4
Midsummer Day	123	13.67	8.83 (7.17–10.48)	2.49 (1.13–3.84)	1.39 (1.14–1.68)	1.28 (1.03–1.57)	28.2/28.6
Midsummer Day ± 14 days	2646	10.14	6.55				
1970–1979							
Reference period^b^	2159	10.90	6.88 (6.59–7.18)	0	1	1	
Midsummer period^c^	832	13.21	8.34 (7.76–8.91)	1.45 (1.17–1.73)	1.21 (1.12–1.31)	1.21 (1.12–1.31)	17.4/17.6
2 days before Midsummer Day	110	14.00	8.84 (7.29–10.38)	1.95 (0.70–3.21)	1.28 (1.07–1.53)	1.36 (1.09–1.67)	22.1/22.1
Midsummer Day ± 14 days	2991	11.46	7.23				
1980–1989							
Reference period^b^	1622	8.19	4.51 (4.28–4.73)	0	1	1	
Midsummer period^c^	622	9.87	5.43 (4.99–5.87)	0.93 (0.71–1.14)	1.14 (1.04–1.24)	1.21 (1.10–1.33)	17.0/17.3
one day before Midsummer Day	111	12.33	6.78 (5.52–8.05)	2.28 (1.24–3.32)	1.52 (1.24–1.82)	1.76 (1.39–2.22)	33.6/43.3
Midsummer Day ± 14 days	2244	8.60	4.73				
1990–1999							
Reference period^b^	989	5.00	2.43 (2.28–2.58)	0	1	1	
Midsummer period^c^	375	5.95	2.89 (2.60–3.18)	0.47 (0.32–0.61)	1.19 (1.06–1.34)	1.20 (1.06–1.35)	16.1/16.4
Midsummer Day	54	6.00	2.91 (2.14–3.69)	0.49 (− 0.14–1.12)	1.20 (0.90–1.56)	1.40 (1.00–1.92)	16.7/28.4
Midsummer Day ± 14 days	1364	5.23	2.54				
2000–2014							
Reference period^b^	1227	3.72	1.70 (1.60–1.80)	0	1	1	
Midsummer period^c^	435	4.24	1.89 (1.70–2.08)	0.19 (0.11–0.28)	1.11 (0.99–1.25)	1.12 (1.00–1.25)	10.2/10.5
Midsummer Day	80	5.33	2.44 (1.88–3.01)	0.74 (0.27–1.21)	1.43 (1.12–1.81)	1.43 (1.09–1.86)	30.3/30.0
Midsummer Day ± 14 days	1662	3.82	1.75				

Risk difference (RD) and crude and adjusted risk ratios (RR) relative to the reference period, and attributable fraction of deaths by day

^a^Adjusted for weekdays and time periods

^b^14 to 4 days before and 4 to 14 days after Midsummer Day

^c^Three days before and three days after Midsummer Day

### Discussion

This study shows that the prominent June peak in mortality from CHD among the working aged population, which has not been reported from other Nordic countries, is an adverse consequence of the Finnish Midsummer festival. Celebrated since pagan times, originally to mark the summer solstice and later to commemorate the feast of St. John, it continues to be a major holiday in Scandinavia. A recent report from Sweden found an increase of acute myocardial infarctions at Midsummer, but the rise was moderate and the association with alcohol consumption remained less substantiated [5]. In Finland, Midsummer is an all-weekend festival marked by travel to lakeside summer cottages, going to sauna, swimming, revelry, eating and very often excessive drinking. During the week preceding Midsummer, alcohol sales triples. As summer holidays often start at Midsummer, there is no obligation to return to work after the festival [4]. In peoples’ minds, Midsummer signifies long, light nights and has a symbolic meaning related to sexuality and fertility.

The most likely explanation for the high mortality from CHD at Midsummer is excessive alcohol consumption, as shown by the successive peaks in fatal alcohol poisonings and coronary deaths on Midsummer Eve and Midsummer Day. The Eastern pattern of drinking which concentrates on weekends and holidays and is also typical of Finland, causes cardiac conduction disturbances, depressed cardiac performance and supraventricular arrhythmias, even in persons without apparent heart disease [6, 7]. In long-time alcohol abusers, acute alcohol ingestion reduces the threshold for ventricular arrhythmias with possibly fatal consequences and may predispose to thrombosis after the drinking binge [8]. Sudden coronary deaths following alcohol ingestion may also occur in people without pre-existing CHD [9]. Acute alcohol consumption also decreases heart rate variability and increase the risk of cardiac death, both in patients with CHD and the general population [10].

Other factors exist that could increase mortality at Midsummer. Sauna bathing is considered safe, even for patients suffering from CHD, but combined with alcohol consumption, it will increase the risk of arrhythmias and sudden death, and together with sweating during long sauna sessions it causes dehydration and haemoconcentration, thus predisposing to thrombosis [11]. The habit of rapid cooling-off in cold water after sauna and doing this repeatedly between the sessions, causes sudden increases in blood pressure and may lead to a rupture of an arterioclerotic plaque, thrombosis and myocardial infarction [12]. In cold water, the simultaneous facial immersion and the cold shock via the skin lead to conflicting chronotropic stimuli from parasympathetic and sympathetic systems, which may trigger arrhythmias and sudden death [13].

Addititional factors include excessive eating, especially salted fish, which easily causes fluid retention among patients suffering from heart failure [14] and could increase the risk of death. Long travels to summer cottages involve physical and emotional triggers that may precipitate acute attacks of CHD [15]. The long geographical distances in this area may prevent timely hospital admission, which may be crucial in case of acute coronary attacks.

The strength of the study is the long time period studied, which allowed an assessment of absolute and relative excess mortalities around Midsummer during the steep decline of CHD since the 1970s. Despite lowering of the absolute mortality attributable to Midsummer, the relative excess remains elevated, as does the preventable fraction of deaths. These trends may have been affected by the increasing number of autopsies and consequent variation in the reliability of death certificates. A limitation of the study is the lack of individual-based data on drinking habits, diet and behavioral patterns during the holiday period. One might argue that the elevated mortality at Midsummer only reflects harvesting of frail individuals who would soon die anyway. This should decrease mortality after the festival, but no such decline was seen here.

The Finnish data show that the risk of adverse cardiac events during holidays can greatly exceed that presupposed by the traditional notion of “holiday heart syndrome” which mainly consists of benign supraventricular arrhythmias following alcohol consumption [6, 7]. The public health gain that could be achieved by prevention is limited by shortness of the risk period, but still an estimated 30% of coronary deaths on Midsummer Day and 10% during the entire festival period are preventable, which is useful information for planning pre-emptive measures at this particular time.

## Data Availability

The R code is available from the author on request.

## Data Availability

The data are confidential and cannot be shared.
